# Dietary food patterns as determinants of the gut microbiome–endocannabinoidome axis in humans

**DOI:** 10.1038/s41598-023-41650-z

**Published:** 2023-09-21

**Authors:** Sophie Castonguay-Paradis, Julie Perron, Nicolas Flamand, Benoît Lamarche, Frédéric Raymond, Vincenzo Di Marzo, Alain Veilleux

**Affiliations:** 1 Centre Nutrition, Santé et Société (NUTRISS), Institut sur la nutrition et les aliments fonctionnels (INAF), Université Laval, Québec, QC G1V 0A6 Canada; 2 Centre de recherche de l’Institut universitaire de cardiologie et de pneumologie de Québec (IUCPQ), Université Laval, Québec, Canada; 3 École de Nutrition, Faculté des sciences de l’agriculture et de l’alimentation (FSAA), Université Laval, Québec, Canada; 4 Département de médecine, Faculté de médecine, Université Laval, Québec, Canada; 5 Joint International Unit between the National Research Council (CNR) of Italy and Université Laval on Chemical and Biomolecular Research on the Microbiome and its Impact on Metabolic Health and Nutrition (UMI-MicroMeNu), Institute of Biomolecular Chemistry, CNR, Pozzuoli, Italy; 6 Canada Research Excellence Chair in the Microbiome-Endocannabinoidome Axis in Metabolic Health (CERC-MEND), Université Laval, Québec, Canada

**Keywords:** Microbiology, Nutrition

## Abstract

The gut microbiota and the endocannabinoidome (eCBome) play important roles in regulating energy homeostasis, and both are closely linked to dietary habits. However, the complex and compositional nature of these variables has limited our understanding of their interrelationship. This study aims to decipher the interrelation between dietary intake and the gut microbiome–eCBome axis using two different approaches for measuring dietary intake: one based on whole food and the other on macronutrient intakes. We reveal that food patterns, rather than macronutrient intakes, were associated with the gut microbiome–eCBome axis in a sample of healthy men and women (n = 195). *N-*acyl-ethanolamines (NAEs) and gut microbial families were correlated with intakes of vegetables, refined grains, olive oil and meats independently of adiposity and energy intakes. Specifically, higher intakes in vegetables and olive oil were associated with increased relative abundance of *Clostridiaceae, Veillonellaceae* and *Peptostreptococaceae*, decreased relative abundance of *Acidominococaceae*, higher circulating levels of NAEs, and higher HDL and LDL cholesterol levels. Our findings highlight the relative importance of food patterns in determining the gut microbiome–eCBome axis. They emphasize the importance of recognizing the contribution of dietary habits in these systems to develop personalized dietary interventions for preventing and treating metabolic disorders through this axis.

## Introduction

Gut microbiota composition and its metabolic activity are increasingly seen as a potential prevention and therapeutic targets to improve cardiometabolic health outcomes of the host. The composition of the gut microbiota is known to be modulated by several factors including, but not limited to, life stages, diseases, environment, and food intake and to have a ubiquitous impact on mostly all human physiology aspects^1^. Even though many associations have been identified between gut microbiota and metabolic health parameters, the exact mechanisms regulating this complex ecosystem remain difficult to identify. The endocannabinoid (eCB) system, a biological system potentially at the interface of the gut microbiota and host metabolism, includes two bioactive lipids derived from arachidonic acid, *N-*arachidonoyl-ethanolamine (anandamide, AEA) and 2-arachidonoyl-glycerol (2-AG), the cannabinoid receptors (CB_1_ and CB_2_) as well as anabolic and catabolic enzymes of these mediators^2^. The extension of the eCB system, the endocannabinoidome (eCBome), includes additional eCB congeners derived from long chain fatty acids, notably within the *N-*acyl-ethanolamines (NAEs) and the 2-monoacyl-glycerols (2-MAGs) families, as well as numerous other enzymes and receptors. This system shares several metabolic functions with the gut microbiota, such as energy metabolism, inflammation, and immunity^3–5^. An increasing amount of evidence points to a bidirectional relationship between gut microbiota activity and the host eCBome system. Of note, germ-free mice harbor altered circulating and tissue levels of eCBome mediators compared to conventionally reared mice, while fecal matter transplant reverse most of these changes^6^. Interestingly, some evidence also highlights the production of endocannabinoid-like mediators by gut bacteria^7–9^.

The presence of eCBome mediators has been shown in virtually all tissues, especially in the brain, immune cells, adipose tissue and gut. These mediators can also be measured in peripheral circulation, where their origin and physiological significance remain uncertain. Circulating levels of eCBome mediators have been strongly associated with BMI^10–12^, NAEs being mainly associated with total fat mass while 2-MAGs seems more closely related to visceral fat mass^13–15^. Moreover, circulating eCBome mediators have also been shown to be associated with the fatty acid intakes in cross-sectional cohorts using dietary recalls as well as in the context of dietary interventions^15–18^. Indeed, a 2-day Mediterranean diet full-feeding intervention, modulates the relative levels of circulating eCBome mediators in a manner reflecting the fatty acid composition of the diet, which means rich in monounsaturated (MUFA) and polyunsaturated (PUFA) fatty acid and poor in saturated fatty acids^15,19^. The profile of dietary fatty acids appeared as a key determinant of the circulating lipidomic profile independently of the increased eCBome tone associated to adiposity. Of note, the Mediterranean diet recommendations are not focused on lipid intakes, but mostly on the consumption of a nutrient rich food such as olive oil, vegetables, fruits, whole grains, legumes, nuts and fish^20^.

We have previously published that a short-term (i.e., 2 days) adherence to the Mediterranean diet recommendations is sufficient to modulate the relative abundance of several gut microbiota taxa^19^ and the circulating endocannabinoidome^15^. However, in a cross-sectional analysis, the macronutrient intake, except fatty acids intake, per se is not related to the circulating endocannabinoidome^15^. We thus hypothesize that whole-diet food pattern, rather than its macronutrient contents, is associated with gut microbiota and the circulating eCBome. We, therefore, aimed here at deciphering the associations between the dietary intake and the gut microbiome–eCBome axis using two different dietary intake approaches measuring whole food and macronutrient intakes. The results obtained provide novel insight on how the gut microbiome–eCBome axis is modulated by the diet and this new knowledge will pave the way to improving the design of future preventive or therapeutic nutritional strategies for metabolic health.

## Methods

### Study cohort

The environment and Microbiota–EndoCannabinoidome Axis (eMECA) cross-sectional clinical trial (NCT03463304) included 102 women and 93 men covering a large range of adiposity phenotypes (Table 1), as described previously ^15^. All recruitment was performed during the winter and spring of the same year. Individuals with enteropathies, alcohol consumption exceeding the Canadian recommendation for men (> 15) and women (> 10 drinks/week), weight change (± 5 kg) in the last 6 months, having taken antibiotics in the last 3 months and pregnant and/or breastfeeding women were not eligible. Participants who consumed cannabis more than once per week or the week preceding the study (n = 5) were excluded, while current tobacco smokers were not excluded (n = 10). Written informed consent was obtained and the project was approved by the Laval University Ethics Committee (2017-328). Detailed data collecting and sampling procedures have been previously described^15^.

**Table 1:** Characteristics of the study sample.

	All subjects		Men (n = 93)	Women (n = 102)
	Mean (SD)	Range	Mean (95% CI)	Mean (95% CI)
Age (year)	41 (17.5)	19–85	42 (38–46)	40 (37–43)
BMI (kg/m^2^)	25.0 (4.7)	13.3–42.0	25.0 (24.6–26.4)	24.6 (23.6–48.2)
Fat mass (kg)	22.0 (10.0)	4.7–54	20.2 (18.4–22.0)	23.6* (21.6–25.6)
Visceral fat mass (kg)	0.7 (0.8)	0–4.3	0.9 (0.7–1.1)	0.5* (0.4–0.6)
Fasting glucose (mmol/L)	5.0 (0.7)	3.7–10.2	5.1 (4.9–5.3)	4.8* (4.7–4.9)
Fasting insulin (pmol/L)	45.6 (30.4)	9.0–188.0	46.6 (40.2–53.0)	44.7 (39.0–50.4)
HbA1c (%)	5.2 (0.4)	4.3–8.2	5.3 (5.2–5.4)	5.2 (5.1–5.3)
Triglycerides (mmol/L)	1.1 (0.6)	0.4–4.5	1.2 (1.1–1.3)	1.0* (0.9–1.1)
Total cholesterol (mmol/L)	4.6 (1.1)	1.8–7.7	4.5 (4.3–4.7)	4.7 (4.5–4.9)
HDL cholesterol (mmol/L)	1.6 (0.5)	0.6–3.3	1.4 (1.3–1.5)	1.8* (1.7–1.9)
LDL cholesterol (mmol/L)	2.5 (0.9)	0.6–5.5	2.6 (2.4–2.8)	2.5 (2.3–4.8)
Total cholesterol/HDL	3.1 (1.0)	1.5–7.7	3.5 (3.3–3.7)	2.8* (2.6–3.0)

*p values < 0.05 compared to mean (t-test).

### Food intakes

Dietary intakes were determined using a self-administered web-based 24 h dietary recall (R24W) validated in this population^21^, as described previously^15^. Food groups were categorized according to the calculation of the Mediterranean food pattern: whole grains, refined grains, fruits, fruit juices, legumes/nuts, vegetables, vegetable juices, olive oil, milk and substitute, fish and sea foods, poultry, eggs, sweets and red meat/processed meat^22^. Coffee and tea consumption were also considered because of purported associations with the gut microbiota and eCBome system.

### Circulating eCBome mediators

Levels of *N*-acylethanolamine (NAEs) and the 2-monoacylglycerol (2-MAGs) were measured using high-performance liquid chromatography coupled to tandem mass spectrometry (LC–MS/MS). The methods^23^ and the circulating levels of the eCBome mediators in this cohort^15^ have been published previously.

### 16S rRNA gene sequencing

Stool bacterial DNA was extracted (QIAGEN, CA, USA), V3–V4 libraries were prepared (Illumina and Axygen Biosciences, CA, USA) and sequences were processed as previously described for this cohort^15^. Diversity indexes were calculated for all samples at an even depth from rarefied OUT table (Vegan R package). For statistical analysis bacterial relative abundances were normalized using Cumulative Sum Scaling (CSS, MetagenomeSeq R package) as specified in the result section and figure legends.

### Statistical analyses

Correlation between dietary intakes, gut microbiota composition and eCBome mediators were computed using Spearman’s rank correlations. Multiple factor analysis (MFA), a dimensionality reduction method tailored to handle multiple groups of variables, was performed with the FactoMineR R package (version 2.4) and the factoextra R package (version 1.0.7). Factors, i.e., groups of variables, included in the MFA model were: Adiposity [fat mass (kg), visceral adipose tissue mass (kg) and BMI (kg/m^2^), n = 3 variables], Metabolic profile [HbA1c (%), fasting glycemia (mmol/L), fasting insulinemia (pmol/L), cholesterol (mmol/L), triglycerides (mmol/L), high-density lipoprotein (HDL) cholesterol (mmol/L), low-density lipoprotein (LDL) cholesterol (mmol/L), n = 8 variables], and Gut microbiota families [CCS-normalized relative abundance of bacterial families in at least 10% of individuals, n = 27 variables). The analysis was performed using families as preliminary analysis reveals that this taxonomic level explains a large part of the gut microbiota variance, provide good statistical power and limit the number of zero. Analysis using genera tended to provide similar results. Circulating levels of eCBome mediators were included in the factors 2-MAGs (n = 7 variables) and NAEs (n = 6 variables). Finally, dietary intakes were included in the Macronutrients [fats (%), proteins (%), carbohydrates (%), fibers (g), alcohol (%), SFA (g), MUFA (g) and PUFA (g], n = 8 variables) and Food groups [See Food intakes section, n = 14 variables] factors. Sex and age were included at first in the model, but these variables didn’t contribute and were not included in the final model. Participants were stratified in two clusters according to the Dimension x* coordinate of each individual.

All analyses were conducted with R software (version 4.1.1). Correlation networks were drawn using ggraph R package (version 2.1.0). GraphPad Prism (version 9.3.1) was used to generate the correlation heatmap and boxplot.

### Ethical approval

All experiments and methods were performed in accordance with relevant guidelines and regulations.

## Results

### MFA of the gut microbiota-eCBome axis

**Figure 1:**
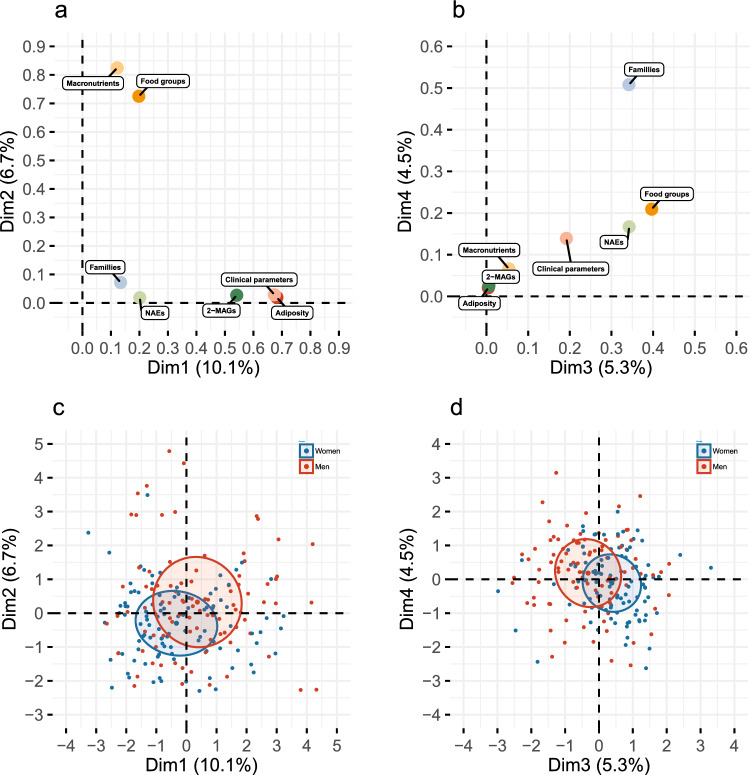
Visualization of the multiple factor analysis (MFA) modeling the gut microbiota eCBome axis parameters with variables of adiposity, clinical parameters, food groups intakes and macronutrients intakes. Graph of factors contribution to (**a**) dimensions 1 and 2, and of (**b**) dimensions 3 and 4 of the MFA model. Graph of individuals of (**c**) dimensions 1 and 2, and of (**d**) dimensions 3 and 4 with sex as factor variables (Women: Blue, Men: Red). Ellipses are standard deviation from the mean center of each group of individuals.

The direct association between circulating eCBome mediators, gut microbiota composition and dietary fatty acid intakes have been previously described in the present cross-sectional cohort as well as in a short-term dietary intervention^15,19^. To decipher the complex interrelation between the gut microbiome–eCBome axis with the diet, we have computed a multiple factor analysis (MFA) model including dietary intakes as two independent factors: one with whole foods (Food groups) and the other with macronutrient intakes (Macronutrients). (Fig. 1). The first four dimensions explain 26.6% of the total variance of the model. Sex was included as a supplementary variable in the MFA model (Fig. 1c and d). Women and men showed a similar distribution in all dimensions of the model and confidence interval ellipse are overlapping.

**Figure 2:**
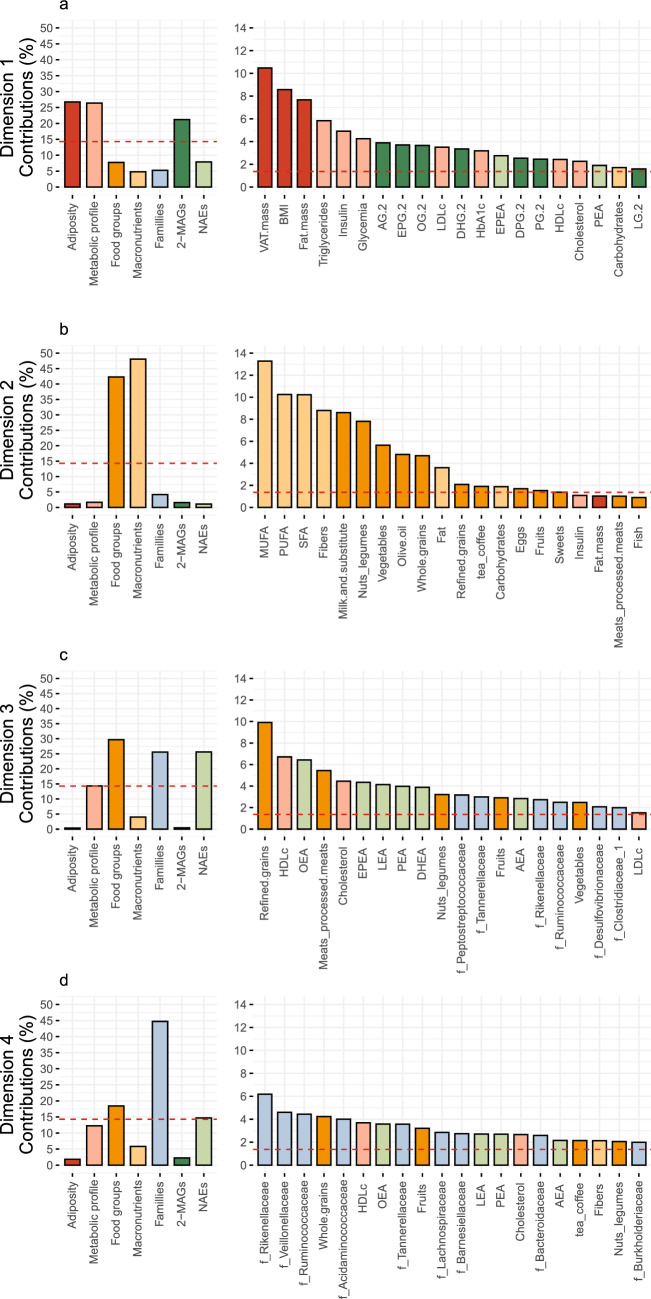
Contribution of variables of the multiple factor analysis (MFA) to (**a**) dimension 1, (**b**) dimension 2, (**c**) dimension 3 and (**d**) dimension 4. Each graph includes the contribution of each factor (Left) as well as the contribution of individual variables (Right). The dashed line corresponds to the expected value if the contribution of each variable was uniform.

The first dimension, which explains 10.2% of the variance, was mostly driven by Adiposity and Metabolic profile variables (Fig. 2a). The circulating levels of 2-MAGs were also associated with the variance of the first dimension, in accordance with previously reported positive correlations with total and visceral fat mass^15^. Concerning the second dimension of the MFA, variables describing food intakes were its only considerable drivers (Fig. 1a and 2b). Overall, this dimension gathered common variances between Food groups and Macronutrients intakes.

Interestingly, circulating levels of all NAE mediators contribute to the dimensions 3 and 4 of the MFA. Adiposity variables were only contributing to the first two dimensions of the model. These observations thus imply that the variance of NAEs explained in the third and fourth dimension of the model is independent of adiposity. Interestingly, the dimensions 3 and 4 were characterized by the contribution of several variables of Gut microbiota and Food groups (Fig. 1b). It is noteworthy, however, that variables within Food groups, but not within Macronutrients, were significant contributors to these dimensions (Fig. 2c and d).

The third dimension gathers the relative abundance of the *Peptostreptococaceae, Tannerellaceae, Rikenellaceae, Ruminoccocaceae, Desulvovibrionaceae* and *Clostridiaceae* families as well as the intakes of refined grains, meats, nuts/legumes, fruits and vegetables (Fig. 2c). Similarly, the fourth dimension included the *Rikenellaceae, Veillonellaceae, Ruminoccoccaceae, Acidaminococcaceae, Tannerellaceae, Lachnospiraceae, Barnesiellaceae, Bacteroidaceae* and *Burkholderiaceae* families as well as the intakes of four food groups, i.e., whole grains, fruits, tea/coffee, and nut/legumes (Fig. 2d). Finally, these dimensions were also associated with total and HDL cholesterol.

In summary, dimensions 3 and 4 of the MFA comprised a subset of variables involved in a complex interrelation between the gut microbiota-eCBome axis and dietary intakes. By design, the model highlights the contribution of these variables while taking into consideration key endogenous variables of these systems, namely adiposity and metabolism. It appears that the relative abundance of gut microbiota families and the circulating levels of eCBome mediators were more closely associated to dietary intake measures based on food groups rather than on specific macronutrients. As previously demonstrated, the associations between macronutrients and mediators of the endocannabinoidome showed virtually no statistical significance^15^. Therefore, subsequent analyses were focused on the contribution of food groups on either system.

### Food groups interrelation with families and circulating NAEs

**Figure 3:**
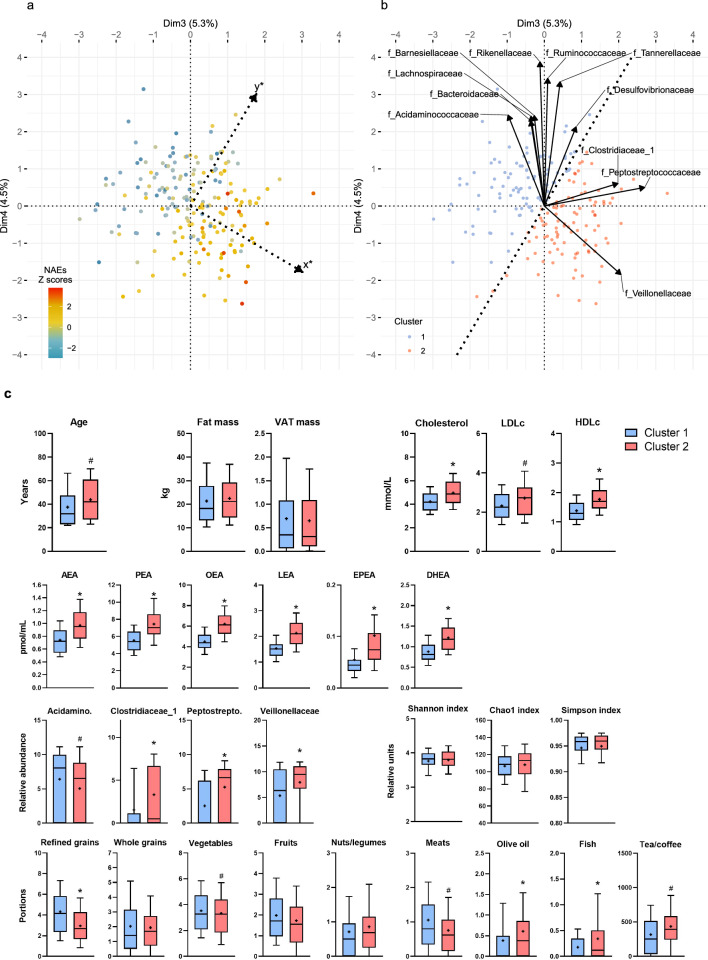
Dimension reduction to optimize the circulating NAEs. (**a**) Original and (**b**) rotated graph of individuals of dimensions 3 and 4. The color gradient represents the mean Z-score of NAEs mediators. The doted vectors correspond to the rotated plan which optimized the variance of NAEs through the new rotated Dimension x*. (**c**) Adiposity, metabolic profile, gut microbiota families, NAEs and dietary intakes (portions for all groups and mL for tea/coffee) of individuals clustered using the dimension x* coordinates. Boxplots include the median, lower/higher quartiles and 1.5 × inter-quartile range whiskers. The means of the distribution is represented by a + sign (n = 97 and n = 98 for clusters 1 and 2 respectively). *Adjusted *p* < 0.05 (Wilcoxon sign rank test with Holm-Bonferroni correction), ^#^*p* < 0.05 before adjustment for multiple comparison (Wilcoxon sign rank test). Holm-Bonferroni corrections have been performed within each group of variables: adiposity and metabolic parameters (n = 11), NAEs and 2-MAGs (n = 13), taxa’s families (n = 27) and food groups (n = 15).

Several food groups and microbial families contribute to both dimensions 3 and 4 and these dimensions explain a certain amount of variance in the circulating levels of NAEs. Variations in circulating levels of NAEs among individuals are highlighted using a color gradient in Fig. 3a. The gradient increases in parallel to the vector x* pointing toward the lower right of the dimensions 3 and 4 planes (Fig. 3a). We computed the vector direction and proceeded with an octagonal rotation of the plane so that the rotated plane optimized the variance of circulating NAEs levels on the Dimension x* (Fig. 3b). The bacterial families contributing the most to dimension x*, and therefore to the variance of circulating NAEs, were *Clostridiaceae*, *Peptostreptococaceae* and *Veillonellaceae*. Other families were more closely following Dimension y* of the rotated plane which was not associated with variances of either NAE or 2-MAG eCBome mediators. To highlight the direction of the association among food groups, families and NAE variables, we stratified the cohort in two clusters according to the Dimension x* coordinate of each individual.

In agreement with the MFA model, individuals in the cluster 2 had significantly higher circulating levels of all NAEs than individuals in cluster 1 (Fig. 3c). Both groups had similar adiposity measures but individuals with higher circulating NAEs levels (i.e., cluster 2) were characterized by higher levels of LDL and HDL cholesterol than individuals in cluster 1. This analysis also highlights that this phenotype (representing by cluster 2) was associated with significantly lower relative abundance of *Acidaminococcaceae* but higher relative abundance of *Clostridiaceae, Peptostreptococaceae* and *Veillonellaceae*. However, these differences were not associated with global differences in gut microbiota composition. Indeed, there was no difference in any of the diversity indexes (i.e., Chao1, Simpson and Shannon) even though some food group intakes (i.e., refined grains and meats) were associated with diversity indexes (Supplementary Table 1). Individuals with high circulating levels of NAEs (cluster 2) reported lower intakes of refined grains and meats but higher intakes of vegetables, olive oil, fish and tea and coffee. These differences in food intakes corresponded to a slightly reduced intake from carbohydrates in cluster 2. Total protein and fat (i.e., total, SFA, MUFA, PUFA) intakes were similar between clusters (Supplementary Fig. S1).

### Network of food groups and the gut microbiota-eCBome axis

**Figure 4:**
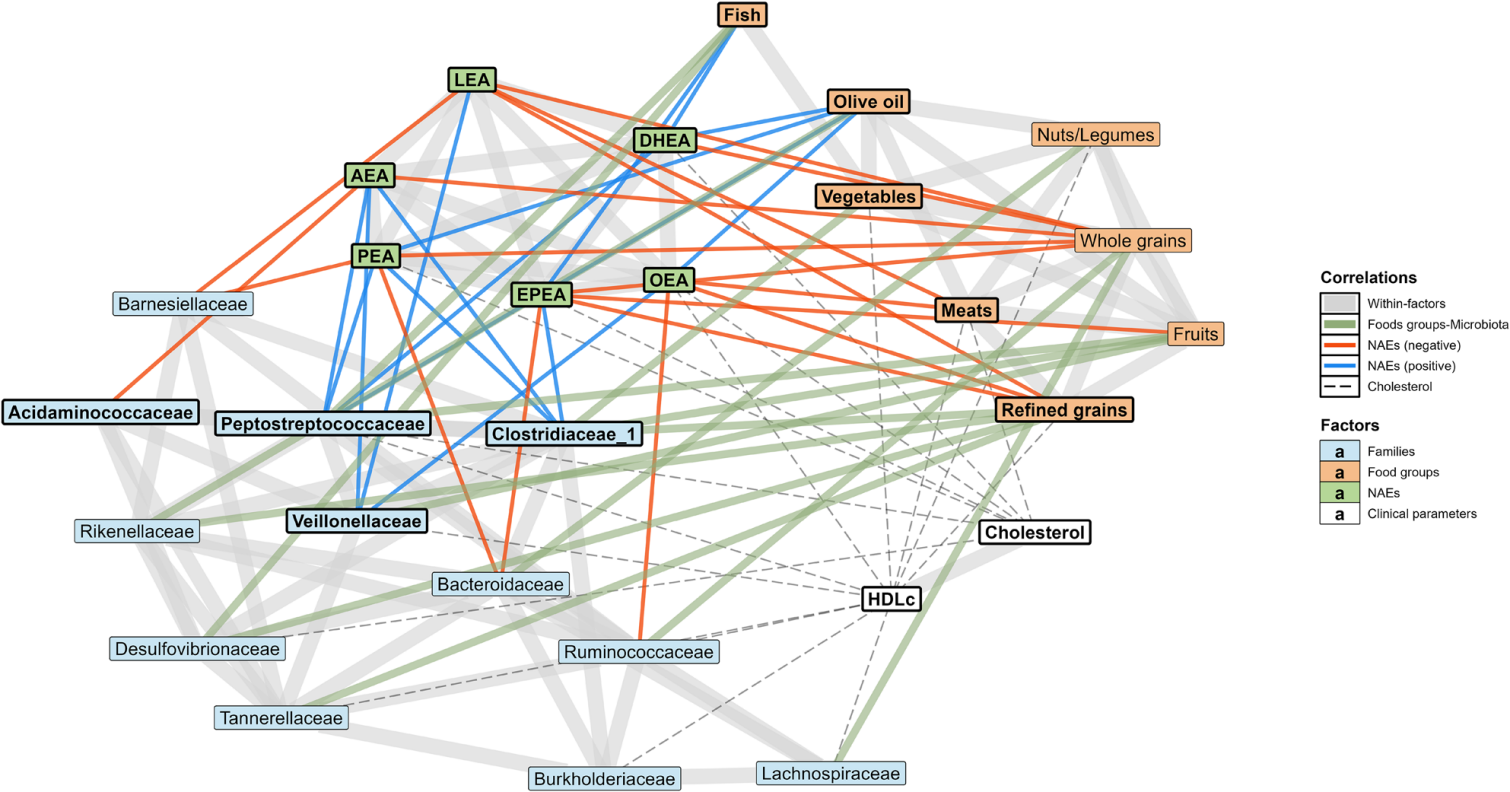
Correlation network of NAEs, gut microbiota families and food groups. Nodes includes all eCBome, microbial families and dietary variables significant contributing to the dimensions 3 and 4 of the MFA. Edges identify significant spearman correlation coefficient between variables (*p* < 0.05, n = 195). Correlations within each variable groups are illustrated by wide grey edges while correlation between food groups and microbial families are indicated by green edges. Only correlation involving NAEs with either food groups or NAEs were colored according to the direction of the correlation (Blue: positive, Red: negative) as indicated in the legend.

To visualize the relationship among the intake of various food groups, gut microbiota and the eCBome, we computed a correlation network (Fig. 4). Dietary intakes of macronutrients were not sufficiently associated with circulating eCBome mediators to enable similar analysis (Fig. 2c,d). The correlation network based on food groups highlighted interactions between the circulating levels of NAEs and food groups and gut microbiota families (Fig. 4 and Supplementary Fig. S2). Moreover, several direct correlations between gut microbiota families and food group intakes were noted. Indeed, the network revealed that the consumption of several food groups was associated with the abundance of specific gut microbiota taxa and with the circulating levels of eCBome mediators. Interestingly, two bacterial families shared an association with the consumption of two or more food groups. *Clostridiaceae* relative abundance was associated with intakes of refined grains, fruits and vegetables, while *Rikenellaceae* was associated with refined grains and olive oil. On the other hand, refined grains and fruits were the food groups with the highest number of correlations with bacterial families.

## Discussion

Dietary intakes are known as a key determinant of the circulating eCBome mediator profile and gut microbiota composition. However, there is still an important knowledge gap in understanding the intricate relationships between these systems and food intake. In a well-characterized cohort of healthy men and women, we have compared two approaches, one based on whole foods and the other on macronutrients, to decipher the association between the diet and the gut microbiome–eCBome axis. To our knowledge, this study is the first to directly compare how intake of different foods and macronutrients explains the interindividual variations in the gut-microbiome–eCBome axis. Moreover, the analysis enabled us to isolate a subset of variables of these two “omes” and of food intakes that share common variance independently of adiposity and other confounding factors affecting these systems. Overall, our analysis suggests that food patterns are more closely related to gut microbiota composition and circulating levels of eCBome mediators than the macronutrient composition of the diet. We revealed that vegetables, refined grains, and olive oil are the main food contributing to circulating NAEs levels, and this specific food pattern is also associated with the relative abundances of the *Clostridiaceae*, *Peptostreptococaceae* and *Veillonellaceae* families in the gut microbiota.

As could be expected, dietary macronutrients content and food groups are highly related variables which, nevertheless, measure distinct aspects of dietary intakes. Consumption of a specific food group could be considered a proxy of specific macronutrient intake, as in the case of, e.g., a diet rich in fruits, vegetables, legumes and whole grains, which is associated with a high fiber intake. However, we demonstrate that macronutrients could not explain as much variance in the gut microbiome composition and the circulating eCBome profiles. Such observation could arise from the fact that food groups, in addition to their macronutrients content, are also associated with differences in other nutrients and in food matrix. The food matrix influences digestion and absorption as well as nutrient availability to the gut microbiota^26^. Moreover, whole foods may surpass the effect of nutrients itself on microbial metabolism and host signaling pathways.

This study takes advantage of food groups based on the Mediterranean diet pattern. Consumption of a diet rich in vegetables, fruits, legumes, nuts, whole grains, MUFA-rich oil and fish has proved to provide many beneficial cardiometabolic effects^27^. We showed that consuming a Mediterranean diet for 48 h alters circulating bioactive lipids, i.e., eCBome and short chain fatty acids^15,19^. Relatively few studies have related changes in gut microbiota composition with the beneficial metabolic effects of this diet. Garcia-Mantrana et al.^28^ showed that the relative abundance of the *Christensenellaceae* family was associated with a higher adherence to the Mediterranean diet and negatively associated with adiposity. Here, increased prevalence of *Christensenellaceae* was associated positively with whole-grain foods consumption and negatively with LEA and PEA circulating levels. However, this association was not observed in the MFA and was not observed in correlations adjusted for fat mass (data not shown). PEA and LEA can respectively bind the G-protein-coupled receptors (GPCR) 55 and 119 and thus improve glucose homeostasis^2^. It remains difficult to predict the overall metabolic impact of changes in the circulating levels of these eCBome mediators because their origin and role in circulation are poorly documented, and each mediator binds different types of receptors to generate a complex cellular response. Nevertheless, as mentioned earlier, this association is aligned with the respective roles on metabolic health of these members of the eCBome^29^.

We highlight novel associations between the relative abundance of the *Peptostreptococcaceae* family with eCBome mediators, such as higher levels of this taxon were observed in individuals with higher circulating levels of all NAEs, apart from OEA. To our knowledge, no potential association has been previously reported between the *Peptostreptococcaceae* family and metabolic health parameters, macronutrients, or whole food consumption. Interestingly, in our analysis this taxon was not directly associated with macronutrient intakes but shared a significant amount of variance with the intakes of food groups and NAEs in the MFA. Indeed, independently of other variables included in the model (i.e., food intakes, adiposity and metabolic profile), the relative abundance of *Peptostreptococcaceae* family was higher in individuals with elevated levels of circulating NAEs.

We observed that *Ruminoccocaceae* relative abundance was associated to dietary intakes of whole-grain foods, from which we can infer a higher intake of fibers. While this taxon was not directly associated with LEA and PEA, these eCBome mediators were negatively associated with the intakes in whole-grain foods.

An association between fruit consumption and *Akkermanciaceae* (i.e. *Akkermansia muciniphila*) relative abundance was found and both variables have been linked to improved metabolic health^31,32^. We previously reported that 2-EPG levels are negatively associated with *Akkermanciaceae* independently of adiposity^15^. However, when including all groups of variables, i.e., dietary intake, adiposity, clinical parameters, *Akkermanciaceae* was not a significant contributor to any dimension of the model.

Numerous studies have already demonstrated that a significant part of the gut microbiota composition variance is explained by the diet^33^, but also by variations in whole food intakes as well as in food patterns^28,34,35^. As the synthesis of different eCB congeners is partly defined by the profile of fatty acids in their phospholipid precursors, we have previously shown that the fatty acid profile of dietary lipids is associated with the profile of the circulating eCBome mediators^15^. The present analysis fills in the knowledge gap of the most potent measures relating dietary intakes and the gut microbiota-eCBome axis. Indeed, here we show that the consumption of specific food groups related to the Mediterranean diet was more closely related to the gut microbiota composition and to the profile of circulating eCBome mediators than intake of specific macronutrients. It should be kept in mind that intakes of food and of macronutrients are closely interrelated. Indeed, we found that higher consumption of olive oil, an important source of oleic acid, is positively associated with circulating levels of OEA, an oleic acid-derived molecule, even though dietary intakes of oleic acid did not significantly correlate with circulating OEA levels^15^. This finding suggests that oleic acid derived eCBome mediators may be more closely related to specific food groups and thus points to the existence of interactions with eating habits related to higher consumption of olive oil. Similarly, circulating levels of AEA and 2-AG were positively associated with consumption of food rich in the eCB precursor arachidonic acid, such as poultry and red meat. However, intake of arachidonic acid was not directly associated with circulating AEA and 2-AG levels^15^. All 2-MAGs congeners were associated with higher consumption of meats, but these associations seem to be secondary to the strong interrelation of these variables with adiposity and the metabolic profile shown in the first dimension of the MFA. Indeed, 2-MAG levels in circulation are strongly associated with visceral adiposity^15^ and so are, on average, meat and processed meat intakes (Supplementary Fig. S3). These independent associations with specific eCBome mediators reinforce the importance of the whole food intake in determining the circulating eCBome mediator profile.

Fish consumption has also been positively associated with some omega-3 eCBome mediators such as EPEA, DHEA and 2-EPG. Overall, fish are rich in the omega-3 fatty acids EPA and DHA, which act as ultimate biosynthetic precursors for these eCBome mediators. Previous studies in mice and humans have demonstrated that fish oil consumption can modify the circulating profile of eCBome mediators, in a way that omega-3 derived mediators are increased, and other mediators are decreased^17,18,36^. This data suggests that the latter mediators are sensitive to the diet, including both dietary intakes of these fatty acids and food groups.

It is worth noting that the first four dimensions of our model explain slightly more than 25% of the variance in these systems. The remaining variance could arise from complex biological interactions, genetic variations, environmental influences, and other intra-individual differences that the model does not capture completely. It is also worth considering that the measured parameters might only represent a single aspect of a biological system (e.g., gut microbiota composition vs gut microbial activity).

This study was carried out in a well-characterized cross-sectional cohort of men and women with a wide range of adiposity and with food intakes monitored thoroughly with a web-based 24 h recall validated for this population. Previously reported correlations were observed between certain food groups and BMI, fat mass and VAT mass (Supplementary Fig. 3), as expected^37–39^. Therefore, comparing both dietary intake measures, i.e., food groups and macronutrients, was an undeniable strength in this study focused on eCBome mediators and gut microbiota, which are emerging players in energy metabolism. Our analysis enabled us to control for the compositional nature of the data as well as for common confounding variables for the two investigated systems, but these results will necessitate further attention to unravel the nature of this relationship in view of the cross-sectional design of this study.

In conclusion, our findings highlight the relative importance of food patterns, compared to macronutrients per se, in determining the gut microbiome–eCBome axis state. It is crucial to consider the presence of such a complex and concomitant relationship between the gut microbiome–eCBome axis and dietary intakes. Indeed, all these systems and the dietary habits are each tightly associated with obesity and metabolic complications. Therefore, our results emphasize the importance of recognizing the contribution of dietary habits in these systems to develop personalized dietary interventions for preventing and treating metabolic disorders through the gut microbiome–eCBome axis.

### Supplementary Information

Supplementary Information.

## Data Availability

Sequencing data for the 16S rRNA sequences were deposited in the NCBI GenBank under BioProject ID PRJNA644138 and under SRA accession number SUB7687442. Individual de-identified subject data, including a data dictionary, related to the analyses included in this manuscript will be made available from the corresponding author on a reasonable request.
