# Supplementary material for: Dietary food patterns as determinants of the gut microbiome–endocannabinoidome axis in humans
Source: Sci Rep. 2023 Sep 21;13:15702. doi: 10.1038/s41598-023-41650-z (PMC10514042; doi:10.1038/s41598-023-41650-z)
Supplement: Supplementary file 1 — Supplementary Information. [file 41598_2023_41650_MOESM1_ESM.pdf]

Dietary food patterns as determinants of the gut microbiome-endocannabinoidome axis in humans

Sophie Castonguay-Paradis, Julie Perron, Nicolas Flamand, Benoît Lamarche, Frédéric Raymond, Vincenzo Di Marzo, Alain Veilleux

Supplementary Table S1

Spearman correlation coefficient between food groups consumption and diversity indexes.

**Food groups**	**Chao1**	**Shannon**	**Simpson**
Whole grains	–	–	–
Refined grains	**-0.23***	**-0.21***	**-0.13***
Vegetable	–	–	–
Fruits	0.12	**0.22***	**0.18***
Nuts and legumes	–	0.13	0.16
Meats and processed meats	-0.12	**-0.14***	**-0.13***
Chicken	–	–	-0.12
Fish	–	–	–
Eggs	–	–	–
Milk and substitute	–	–	–
Olive oil	–	–	–
Vegetable and fruits juice	–	–	–
Sweets	–	–	–
**Macronutrients**			
Carbohydrates	–	–	–
Fat	–	–	–
SFA	–	–	–
MUFA	–	–	–
PUFA	**-0.13***	–	–
Proteins	–	–	-0.12
Alcool	–	–	–
Fibers	0.11	–	–

– Spearman Rho correlation coefficient with p values; * $p<0.05$

Dietary food patterns as determinants of the gut microbiome-endocannabinoidome axis in humans

Sophie Castonguay-Paradis, Julie Perron, Nicolas Flamand, Benoît Lamarche, Frédéric Raymond, Vincenzo Di Marzo, Alain Veilleux

Supplementary Figure S1

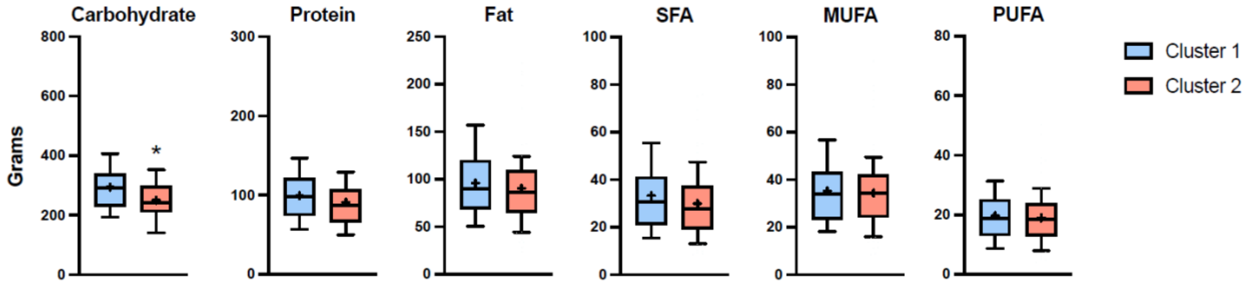

Supplementary Figure 1: Macronutrient intakes according to clusters. Boxplots include the median, lower/higher quartiles and 1.5x inter-quartile range whiskers. The means of the distribution is represented by a + sign. * Indicate significant differences between clusters using Student's t-test or Wilcoxon sign rank test ($p < 0.05$, n=97 and n=98 for clusters 1 and 2 respectively).

Dietary food patterns as determinants of the gut microbiome-endocannabinoidome axis in humans

Sophie Castonguay-Paradis, Julie Perron, Nicolas Flamand, Benoît Lamarche, Frédéric Raymond, Vincenzo Di Marzo, Alain Veilleux

Supplementary Figure S2

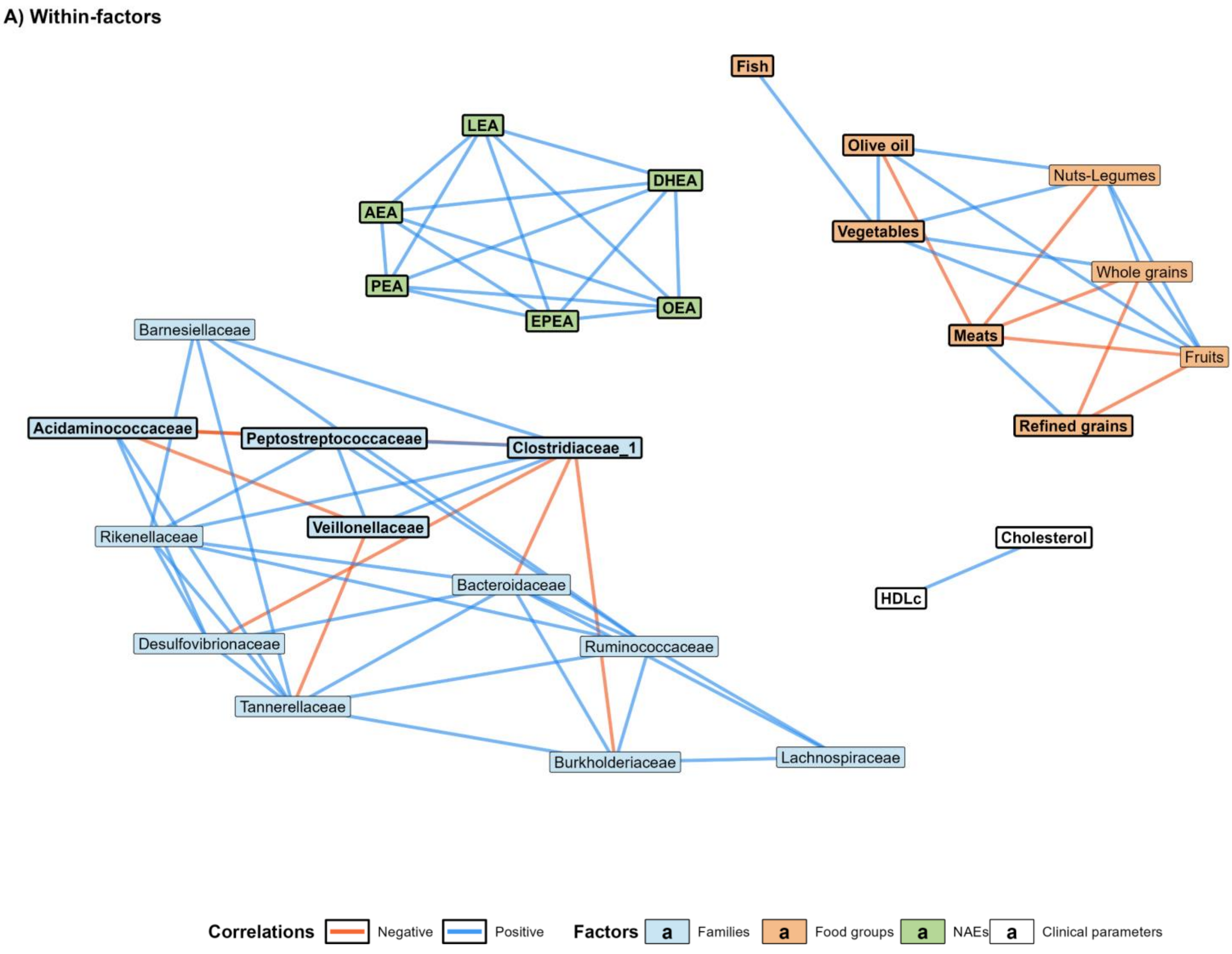

Dietary food patterns as determinants of the gut microbiome-endocannabinoidome axis in humans

Sophie Castonguay-Paradis, Julie Perron, Nicolas Flamand, Benoît Lamarche, Frédéric Raymond, Vincenzo Di Marzo, Alain Veilleux

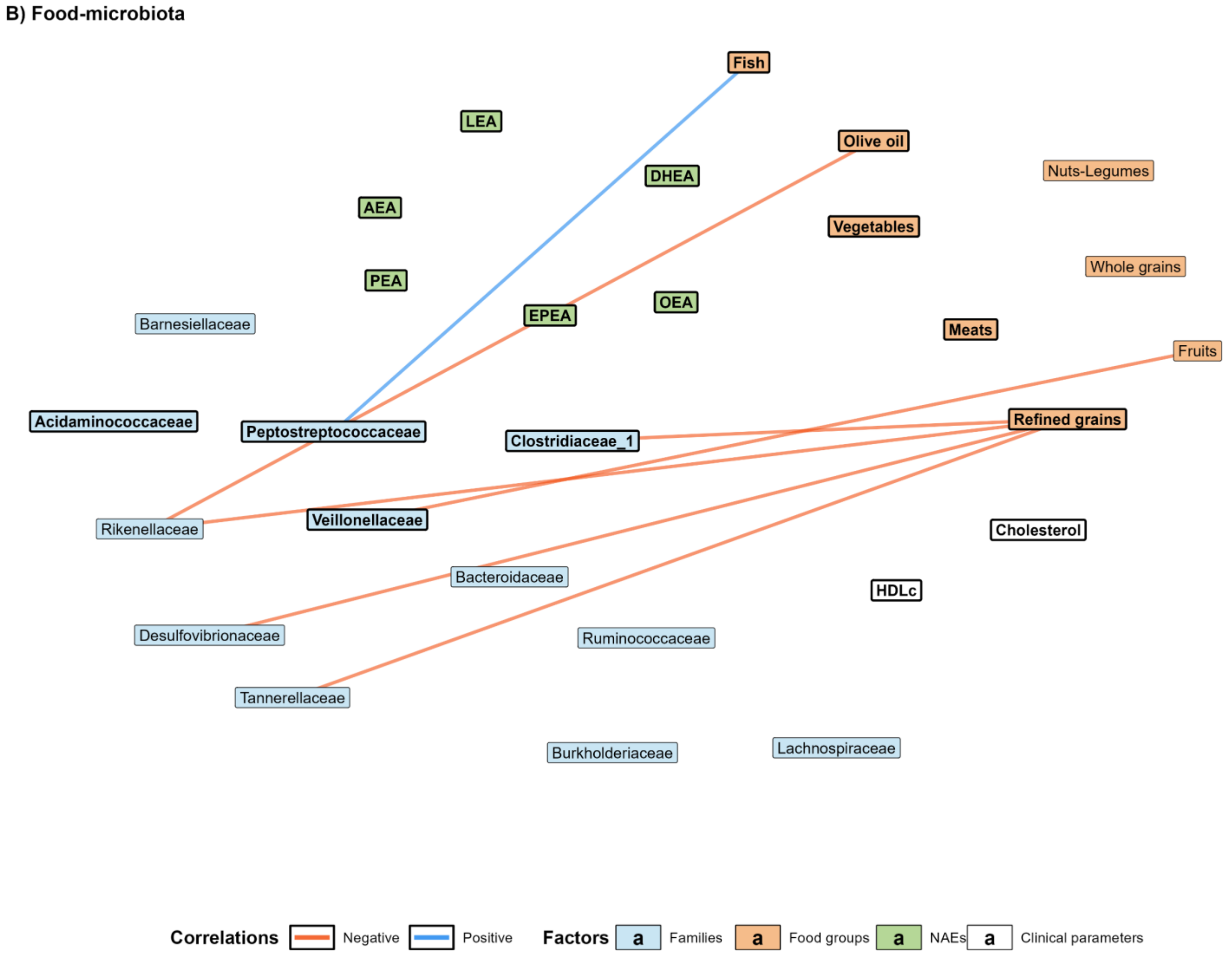

Dietary food patterns as determinants of the gut microbiome-endocannabinoidome axis in humans

Sophie Castonguay-Paradis, Julie Perron, Nicolas Flamand, Benoît Lamarche, Frédéric Raymond, Vincenzo Di Marzo, Alain Veilleux

C) NAEs

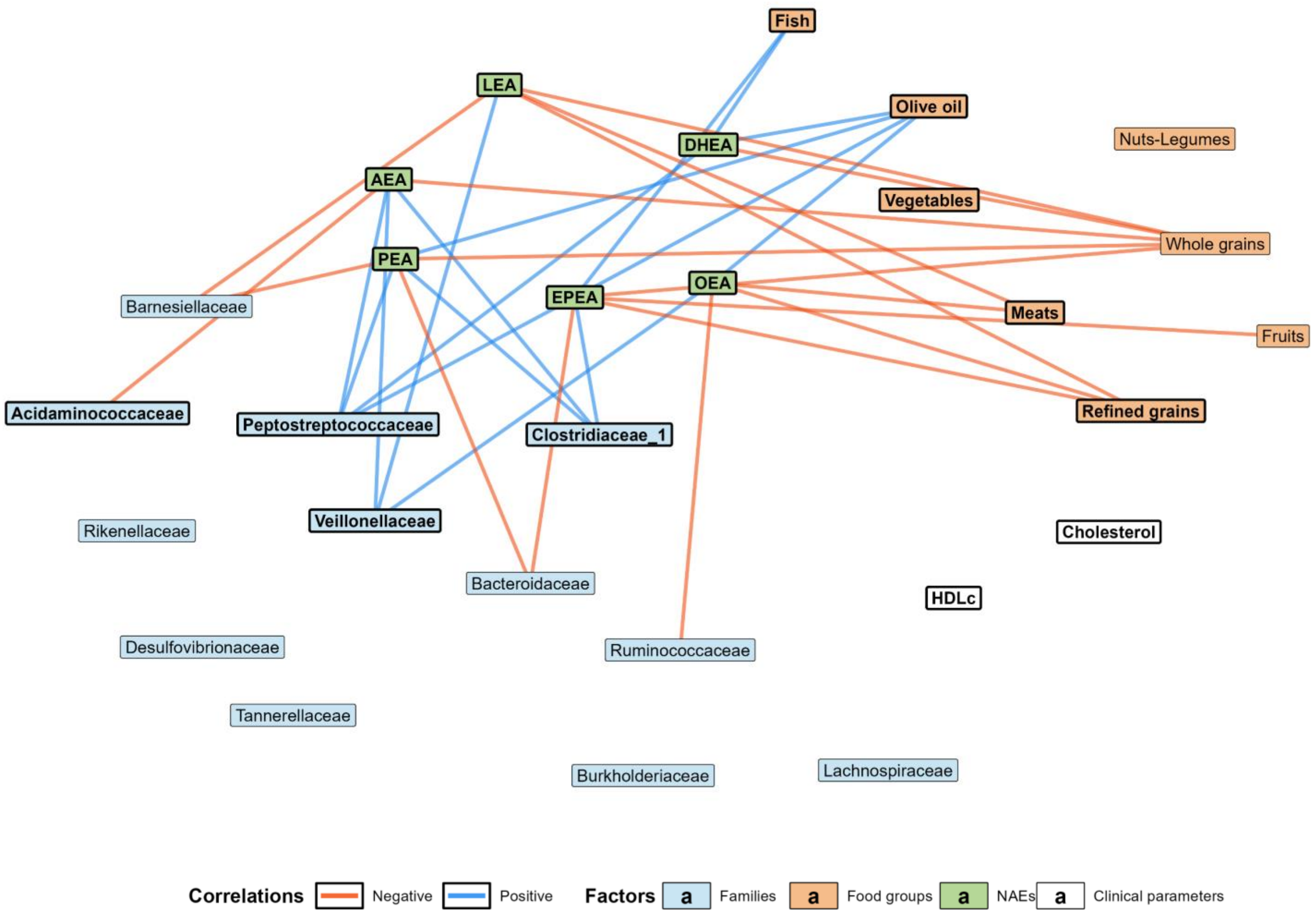

Dietary food patterns as determinants of the gut microbiome-endocannabinoidome axis in humans

Sophie Castonguay-Paradis, Julie Perron, Nicolas Flamand, Benoît Lamarche, Frédéric Raymond, Vincenzo Di Marzo, Alain Veilleux

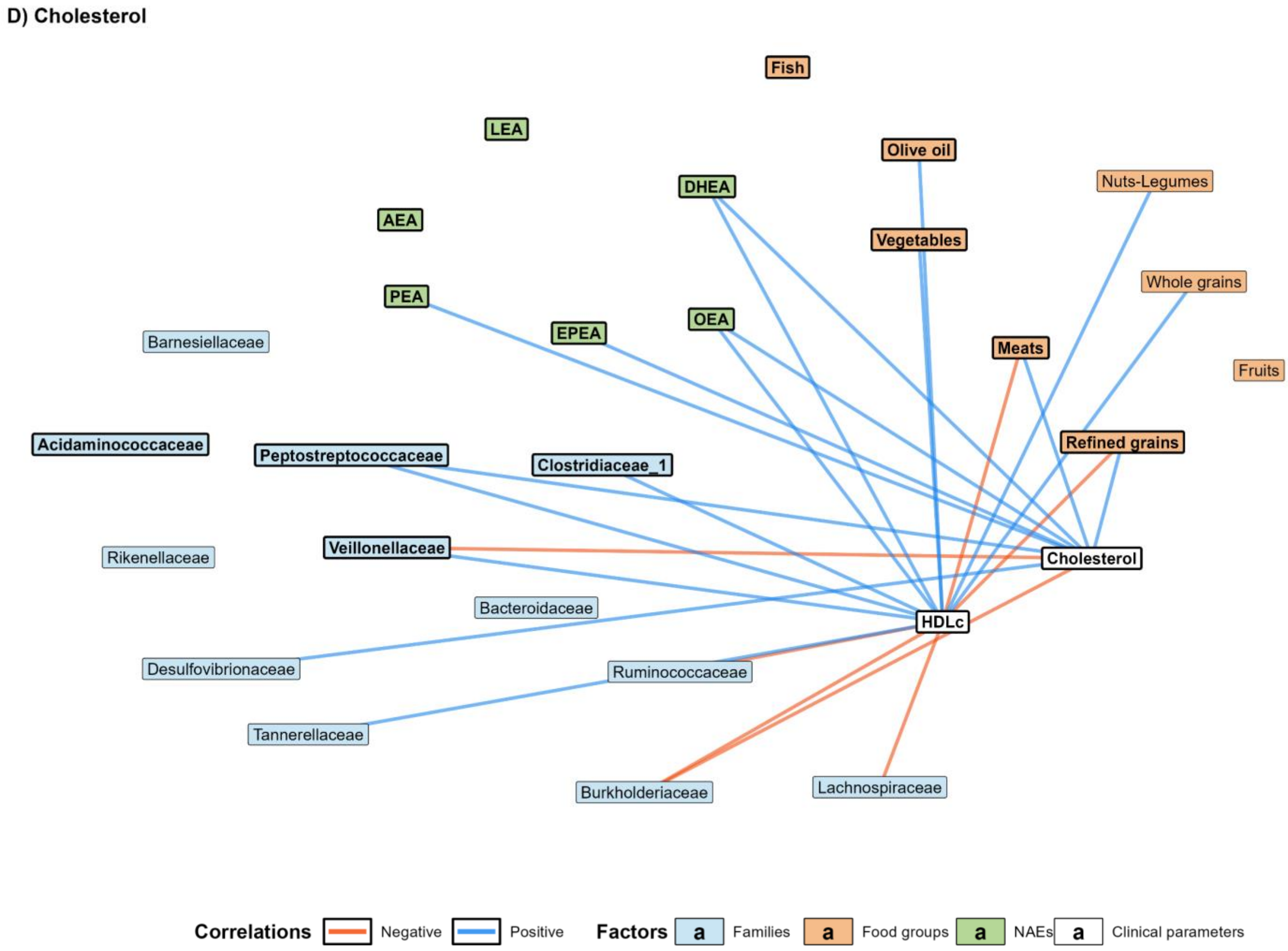

Supplementary Figure 2: Correlation network of NAEs, gut microbiota families and food groups. The figure highlights A) Within-factors, b) Food and Microbiota, C) NAEs and Microbiota or Foods, and D) Cholesterol-related correlations of the network. Nodes includes all eCBome, microbial families and dietary variables significant contributing to the dimensions 3 and 4 of the MFA. Edges identify significant spearman correlation coefficient between variables (p <0.05, n=195). Correlations were colored according to the direction of the correlation (Blue: positive, Red: negative) as indicated in the legend.

Dietary food patterns as determinants of the gut microbiome-endocannabinoidome axis in humans

Sophie Castonguay-Paradis, Julie Perron, Nicolas Flamand, Benoît Lamarche, Frédéric Raymond, Vincenzo Di Marzo, Alain Veilleux

Supplementary Figure S3

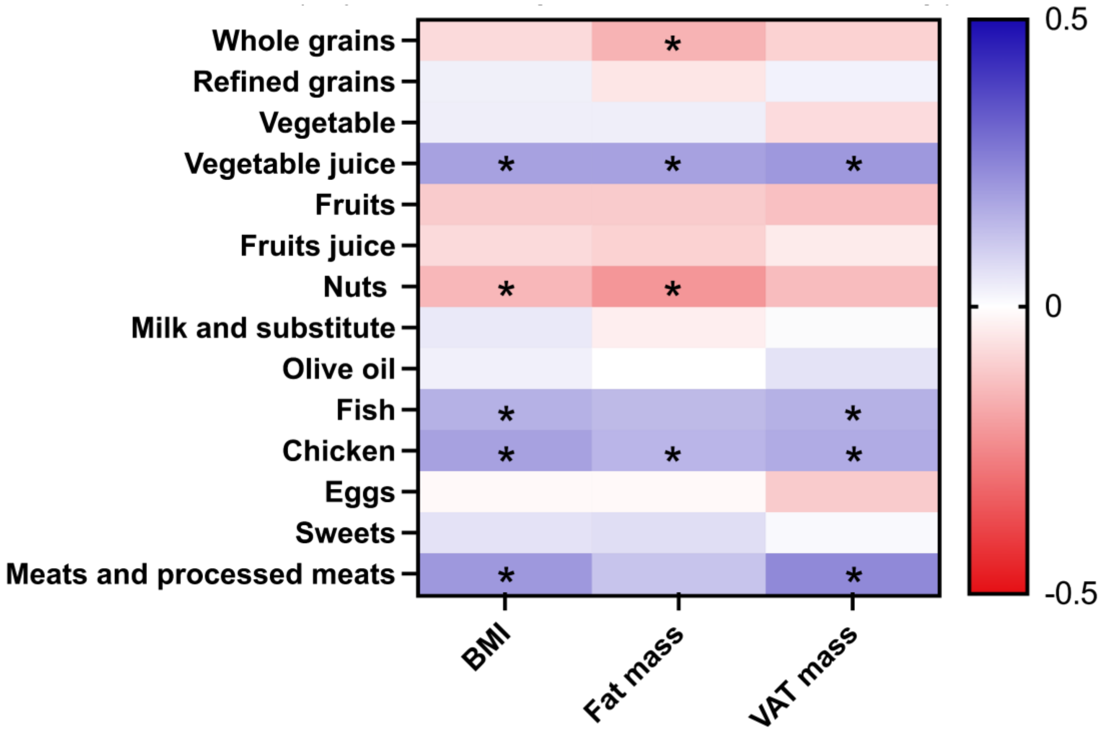

Supplementary Figure 3: Correlation heatmap between food groups and adiposity/anthropometric measures. Spearman's rho values are represented in blue and red as indicated in the figure legend. * Indicated significant correlation ($p<0.05$, $n=195$)
